# Environmental Factors Driving Seed Hydration Status of Soil Seed Banks and the Implications for Post-fire Recruitment

**DOI:** 10.3389/fpls.2021.795003

**Published:** 2022-01-05

**Authors:** Ryan Tangney, David J. Merritt, Ben P. Miller

**Affiliations:** ^1^Centre for Ecosystem Science, School of Biological, Earth and Environmental Sciences, University of New South Wales, Sydney, NSW, Australia; ^2^Kings Park Science, Biodiversity and Conservation Science, Department of Biodiversity, Conservation and Attractions, Kings Park, WA, Australia; ^3^UWA School of Agriculture and Environment, The University of Western Australia, Perth, WA, Australia; ^4^School of Biological Sciences, University of Western Australia, Crawley, WA, Australia

**Keywords:** seed survival, seed hydration, fire season, seed water activity, seed banks

## Abstract

Changes in fire regimes due to climate change and fire management practices are affecting the timing, length, and distribution of vegetation fires throughout the year. Plant species responses and tolerances to fire differ from season to season and are influenced by species-specific phenological processes. The ability of seeds to tolerate extreme temperatures associated with fire is one of these processes, with survival linked to seed moisture content at the time of exposure. As fire is more often occurring outside historic dry fire seasons, the probability of fire occurring when seeds are hydrated may also be increasing. In this study, we set out to understand the seasonal dynamics of seed hydration for seeds of Banksia woodland species, and how certain seed traits interact with environmental conditions to influence survival of high temperatures associated with fire. We measured the moisture content of seeds buried to 2 cm in the soil seed bank for four common native species and one invasive species on a weekly basis throughout 2017, along with soil moisture content and environmental correlates. We determined water sorption isotherms at 20°C for seeds of each species and used these functions to model weekly variation in seed water activity and predict when seeds are most sensitive to soil heating. Using Generalised additive models (GAMs), we were able to describe approximately 67% of the weekly variance in seed water activity and explored differences in seed hydration dynamics between species. Seed water activity was sufficiently high (i.e., ≥ 0.85 *a*_w_) so as to have created an increased risk of mortality if a fire had occurred during an almost continuous period between May and November in the study period (i.e., 2017). There were brief windows when seeds may have been in a dry state during early winter and late spring, and also when they may have been in a wet state during summer and late autumn. These data, and the associated analyses, provide an opportunity to develop approaches to minimize seed mortality during fire and maximize the seed bank response.

## Introduction

Post-fire recruitment from seeds is a fundamental mechanism for the persistence, regeneration, and expansion of plant populations in fire-prone ecosystems (Pausas and Keeley, 2014). How plant species respond to, and tolerate the effects of, fire modulates through seasons (Savadogo et al., 2012; Miller et al., 2019, 2020a,b) under the influence of local environmental conditions and seasonal phenological patterns of the species within the ecosystem (Miller et al., 2020a, 2021; Tangney et al., 2020a).

Soil seed banks are the primary repository for seeds within many ecosystems (Thompson, 1987; Saatkamp et al., 2014). The ability of seeds in the soil to persist through fire and sense their environment to time germination, and consequently seedling emergence, to coincide with periods of the year when conditions are most suitable for seedling survival is key to successful regeneration (Baskin and Baskin, 2001). Seeds close to the soil surface have the highest chance of germination and emergence, yet they are also most exposed to the high temperatures generated by fire, where soil temperatures can exceed 200°C in the upper 2 cm (Stoof et al., 2013; Tangney et al., 2018). Soil temperatures during fire moderate with depth due the insulating properties of soil (Tangney et al., 2020a), increasing the chances of survival for seeds buried deeper within the soil. However, increased moisture content of the seeds at the time of exposure to fire temperatures strongly reduces the chances of seed survival (Fer and Parker, 2005; Ruprecht et al., 2016; Tangney et al., 2019).

Seeds with permeable seed coats that reside within the soil seed bank cycle between hydrated and dry states according to the surrounding soil moisture conditions (Turner et al., 2006), and patterns of seed hydration within the soil may be influenced by a range of weather conditions and their influence on the soil conditions (e.g., the amount of rain, prevailing temperatures, or the evaporation rate). Following rainfall, dry seeds passively imbibe water due to the high-water potential gradient between the seeds and their surrounds (Hegarty, 1978; Wuest, 2007). Seeds can remain in a non-germinated but viable state, with moisture content fluctuating seasonally, until environmental conditions are suitable for germination, or until loss of viability (Long et al., 2015).

Seed moisture content drives physiological processes within seeds, and within the soil seed bank, with different physiological processes becoming possible as moisture content varies with changing environmental conditions (Long et al., 2015). For instance, metabolic and cellular processes engage at different thresholds of seed water potential, or water activity, as seeds hydrate (Walters and Engels, 1998; Walters et al., 2005). These thresholds of water activity can be described by constructing water sorption isotherm functions that depict the relationship between seed moisture content and equilibrium relative humidity (at a given temperature; Walters, 2004). The relationship between moisture content and relative humidity is non-linear reflecting the differing properties of water and water activities as seed moisture content increases (Walters and Engels, 1998). Key thresholds of water activity have been defined by Walters et al. (2005): metabolic processes in seeds are greatly limited with water contents in equilibrium with <15–20% RH (hydration level I, <0.15–0.2 water activity *a*_w_), while 85% RH is the threshold for hydration level III, where metabolic processes including respiration become active. In the context of seed survival through the passage of fire, lethal temperature thresholds of seeds are higher at seed moisture contents below region III of the isotherm (i.e., <85% RH). In these drier seeds, measured lethal temperatures exceed 120°C, and can reach as high as 150°C (Tangney et al., 2019). Seeds hydrated to region III (i.e., ≥85% RH or 0.85 *a*_w_) have a significantly reduced tolerance to high temperatures, with lethal temperature thresholds reduced to between 50 and 100°C (Tangney et al., 2019). The risks of seed mortality during fire are therefore higher outside of periods of low soil moisture when seeds are dry.

Changing fire regimes, including shifts in fire season, are motivating improved understanding of mechanisms of plant responses and recovery to fire in different seasons (Miller et al., 2019). In this study, we sought to understand the seasonal dynamics of seed hydration status in relation to prevailing soil and weather conditions in order to identify periods of higher risk of fire temperatures exceeding seed survivability. Our study was conducted within the southwest of Western Australia, which has a hot-summer mediterranean-type climate and unique and highly diverse plant species and communities. Banksia woodlands occur across the Swan Coastal Plain and this vegetation type has recently been classified as a threatened ecological community, due to climate change, invasive species modified fire regimes, and land clearing for urban development, resulting in less than 60% of the original extent of Banksia woodlands currently remaining (Department of the Environment, 2018). Because the current distribution of Banksia woodlands is comprised of remnant fragments, many within and around the Metropolitan area of Perth, local land managers are facing an increasing challenge managing bushfire risk to protect significant economic, cultural, and ecological values in and around Banksia woodlands (Ritchie et al., 2021).

Banksia woodlands occur within a Mediterranean climate, which produces a strongly seasonal climate. Rainfall is generally confined to the late autumn through to early spring (May – September), in which time ~75% of the annual rainfall occurs (Bureau of Meteorology, 2020). While the natural wildfire season is confined to the period of the year when surface and fine fuels are driest, in summer and autumn (November–April; Plucinski (2014)). Options for the management of the fire risk in biodiverse woodlands across the southwest of Western Australia include the use of hazard reduction burns, where fires recur at approximately 10–20-year interval, with few areas remaining unburned for >50 years. The historical fire season is likely summer-autumn (Ritchie et al., 2021), whereas to reduce the intensity of the fire and the risks of uncontained fire events, prescribed burning occurs during cooler and wetter months of March–November (Burrows and McCaw, 1990), predominately during spring months (August–November), which may affect the persistence and recovery of some species following fire (Miller et al., 2020; Tangney et al., 2020a,b; Miller et al., 2021). Further, surface fuels that contribute to soil heating can exceed an average of 10 Mg/ha^−1^ within 10 years following fire and exceed 15 Mg/ha^−1^ in longer unburnt areas of Banksia woodlands (Tangney et al., 2021) which may be sufficient to yield soil temperatures that exceed lethal temperature thresholds in some seeds (Tangney et al., 2020a).

Consequently, the timing of these introduced fires potentially overlaps with periods during which seeds that are within soil seed banks are hydrated and lethal temperature thresholds are lower, with many species forming persistent seed banks, where a large proportion of seeds reside within the top 5 cm of soil (Rokich et al., 2016). Within this context, we measured and modelled the hydration status (specifically seed water activity, *a*_w_) of seeds placed in the soil seed bank over the course of 1 year. Using seeds from five species common in Banksia woodlands, which produce seeds that will be present within soil seed banks at periods when fires may occur, our aim was to quantify the role of soil moisture content and local weather patterns including rainfall and evaporation rate in driving patterns of seed hydration. We focused on identifying periods of seed hydration above and below critical thresholds that are known to influence lethal temperatures for our study species. This allowed us to examine the seasonal variation in seed hydration and infer risks of seed mortality during aseasonal fire.

## Materials and Methods

### Study Species

We assessed variation in seed moisture content in the soil over a 1-year period in five species common in Banksia woodlands: *Anigozanthos manglesii*, *Asparagus asparagoides*, *Banksia prionotes* (weakly serotinous), *Banksia sessilis* (weakly serotinous), and *Conostylis candicans* (Table 1). All species, except *A. asparagoides*, are native to Banksia woodlands. Mature seeds of each of the four native species were collected from a minimum of 10 plants within wild plant populations from remnant Banksia woodland fragments within the Perth region during 2015. These species were selected as representative of common Banksia woodlands species that release mature seeds, which readily imbibe water (i.e., seeds do not possess physical dormancy). We included two serotinous species, on the basis that both species are weakly serotinous within the Perth region and readily release mature seeds from their fruits, without need for heat to induce follicle opening (Cowling and Lamont, 1985). Seeds of all species thus commonly reside within the soil seed bank or on or near the soil surface during times of fire. We included *A. asparagoides* which is a common weed species within Banksia woodlands to assess whether native and non-native species were differentiated in their hydration dynamics in a way that might alter their risk of mortality. Following the collection and cleaning of seeds, each seed batch was stored at 15°C and 15% RH prior to use in experiments. To ensure filled seeds were used for experiments, seeds from each species were X-rayed using a Faxitron Specimen Radiography System (MX-20 Cabinet X-ray Unit; Faxitron, Wheeling, IL, United States) and any non-filled seeds were discarded.

**Table 1:** Species used for this analysis and their key attributes.

Species	Family (aceae)	Seed storage syndrome	Dormancy Class	Native	Seed weight (mg)	*T*_50w_ (°C)	*T*_50d_ (°C)
*Anigozanthos manglesii*	Haemodor	Soil	MPD	Yes	1.0	83.5	123.8
*Asparagus asparagoides*	Asparag	Soil	PD	No	6.5	72.2	75.5
*Banksia prionotes*	Prote	Weakly serotinous	ND	Yes	23.5	94.2	131.6
*Banksia sessilis*	Prote	Weakly serotinous	ND	Yes	6.2	98.5	144.0
*Conostylis candicans*	Haemodor	Soil	PD	Yes	0.3	114.6	130.5

*Native data sourced from FloraBase (Western Australian Herbarium, 1998. Florabase), Seed Weight, and T50 values sourced from (Tangney et al., 2019). T_50_ values represent temperatures at which 50% of the seed population is killed. T_50w_ values are presented for wet seeds (> 0.85 a_w_), and T_50d_ for dry seeds (0.5 a_w_) at 20°C. Dormancy classes: MPD refers to seeds with Morphophysiological dormancy, PD refers to seeds with Physiological dormancy, and ND refers to seeds with No dormancy*.

### Construction of Moisture Sorption Isotherms

Water sorption isothermic functions describe the functional thresholds of water activity within seeds and depict the relationship between seed moisture content and equilibrium relative humidity (at a given temperature). To construct water sorption isotherms, three replicate samples of seeds from each of the five species were first placed inside small, open paper envelopes. The number of seeds per replicate within each envelope was 50 for *A. manglesii*, 20 for *A. asparagoides*, 20 for *B. prionotes*, 20 for *B. sessilis*, and 200 for *C. candicans*. Envelopes were placed inside air-tight polycarbonate electrical enclosure boxes (28 cm × 28 cm × 14 cm; NHP Fibox, Australia), suspended above non-saturated solutions of LiCl, with the concentration of LiCl within each box adjusted to achieve the desired relative humidity conditions of 15, 20, 30, 50, 70, 80, 90, and 95% RH (741, 640, 520, 364, 237, 171, 94, and 48 g L^−1^ of LiCl, respectively; anhydrous, Sigma®, Australia; Hay et al., 2008). To achieve 10% RH, a saturated LiCl solution was used, and to attain 5% RH a saturated ZnCl_2_ solution was used (Vertucci and Roos, 1990). All boxes were then placed inside an incubator at 20°C. After 3 weeks at respective storage conditions, seeds were retrieved and weighed, before being dried in an oven (Contherm, Korokoro, New Zealand) for 17 ± 1 h at 103°C (International Seed Testing Association, 2021). Seed moisture content was determined gravimetrically on a dry weight basis.

### Measuring Seed Moisture Content of Soil Stored Seeds

Filled seeds from each species were partitioned into 153 nylon mesh bags (holes 2 μm in size), in order to retrieve from the soil three replicate bags for each species each week, for 51 weeks. The number of seeds placed into each bag was varied according to seed weight (Table 1) to achieve approximately 1 g dry weight per replicate. For *A. asparagoides*, *B. prionotes* and *B. sessilis* there was 17 seeds per bag, for *A. manglesii* 34 seeds per bag, and for *C. candicans* 167 seeds per bag.

Bags containing viable seeds and a small amount of bleached white sand were placed into shallow black seedling trays, with three replicate trays per species. On the 2/1/2017 trays were placed next to each other along the edge of a closed (to public access) sand track within bushland of Kings Park, Perth: like most Banksia woodlands, soil within Kings Park is grey sand. A shallow hole was dug below trays to ensure there was soil contact with the base of the tray. Two centimetres of soil was placed on top of each of the trays, as well as a thin layer of leaf litter, similar to the surrounding undisturbed soil. The 15 trays were buried in groups of five (one species per tray), with trays within groups randomly positioned and each group of five trays spaced 5 m apart.

For each species, in every week for 51 weeks, one remaining seed bag was removed from each replicate tray, placed into an airtight resealable plastic bag and taken immediately back to the laboratory at Kings Park for weighing. Seeds were removed from the nylon mesh bags, separated from any sand and each replicate batch of seeds from each species was weighed separately, before being transferred to a clear plastic container for drying, and seed moisture content was determined gravimetrically after drying seeds at 103°C for 17 ± 1 h.

### Measuring Soil Moisture Content

Three replicate soil samples were collected weekly, adjacent to each seed tray using an 8 cm deep × 2 cm diameter plastic cylinder. Each soil sample was placed in an airtight bag before being taken directly for weighing. Soils were weighed in the cylinder and then oven (Contherm, Korokoro, New Zealand) dried at 103°C for a minimum of 17 h. Soil moisture was calculated following ((dry soil weight - wet soil weight)/dry soil weight x 100). Cylinder weight was removed from both wet and dry samples.

### Weather Data

Weather data for each day of 2017 were obtained from the Bureau of Meteorology (2020) Perth Metro weather station (Lat: −31.92, Lon: 115.87, elevation: 25 m–Station ID: 009225.); the closest Bureau of Meteorology weather station to the seed burial area (approximately 6 km away). A range of weather variables were collated throughout 2017, all of which were averaged over the 7 days prior to each respective seed collection date. Weather variables included mean maximum temperature, mean daily relative humidity, mean daily pan evaporation, mean daily rainfall, and the sum of weekly rainfall. From the rainfall records, the number of days since last rain >1 mm (DSLR) was also calculated.

### Data Analysis

Isothermal functions were plotted from fitted third degree polynomials for each species. Due to the nature of the cubic polynomial function, estimates above 100% relative humidity were limited to 99.9% relative humidity to align with the upper most water activity zone (i.e., >0.99 *a*_w_; Walters et al., 2005). Based upon the measured moisture content of the seeds retrieved from the soil, seed water activity (*a*_w_ = RH/100) was determined *via* the third degree polynomial functions. With risks of seed mortality during fire being greatly increased upon hydration of seeds to above 0.85 *a*_w_ (Tangney et al., 2019), we focused subsequent analyses on seed moisture contents in terms of water activity and identifying periods of the year when seed moisture contents were above or below this threshold of water activity (i.e., the boundary between hydration level II and III; Walters, 2004).

### Generalised Additive Models

To model the influence of weather variables as drivers of seed water activity (*a*_w_) in the soil and understand the environmental requirements for seeds to become “wet” (i.e., moisture contents equivalent to above 0.85 *a*_w_), we engaged a model selection approach, commencing with full subsets generalised additive modelling (GAM) *via* the FSSgam package in R (Fisher et al., 2018). This approach allowed us to construct a set of credible models from a full variable set (mean maximum temperature, mean daily relative humidity, mean daily pan evaporation, mean daily rainfall, and the sum of weekly rainfall, days since last rain >1 mm and mean soil moisture) and compare these using Akaike’s Information Criterion corrected for small sample size (AICc) and AICc model weights (ωAICc), which represent the probability or weight of evidence for each model (Hurvich and Tsai, 1989; Burnham et al., 2011). Models with ΔAICc < 2 were considered as having substantial support (Burnham and Anderson, 2004). Seed water activity was modelled using the gam () function in the mgcv package in R (Wood and Wood, 2015) using a beta (logit link) distribution. All interactions between variables within the model set were allowed. The smoothing parameter was limited to a simple spline (*k* = 3) to avoid overfitting. The Pearson’s correlation cut-off in FSSgam was limited to 0.28 to avoid correlated predictors. Variance explained for GAMs was estimated as adjusted *R*^2^ as reported by gam ().

All statistical analyses were undertaken in the program R (ver 4.0.3, R Core Team, 2020), and visualisations were completed using the ggplot2 (Wickham, 2011), gridExtra (Auguie et al., 2017), ggpubr (Kassambara and Kassambara, 2020), and cowplot (Wilke et al., 2019) packages.

## Results

### Moisture Sorption Isotherms

**Figure 1:**
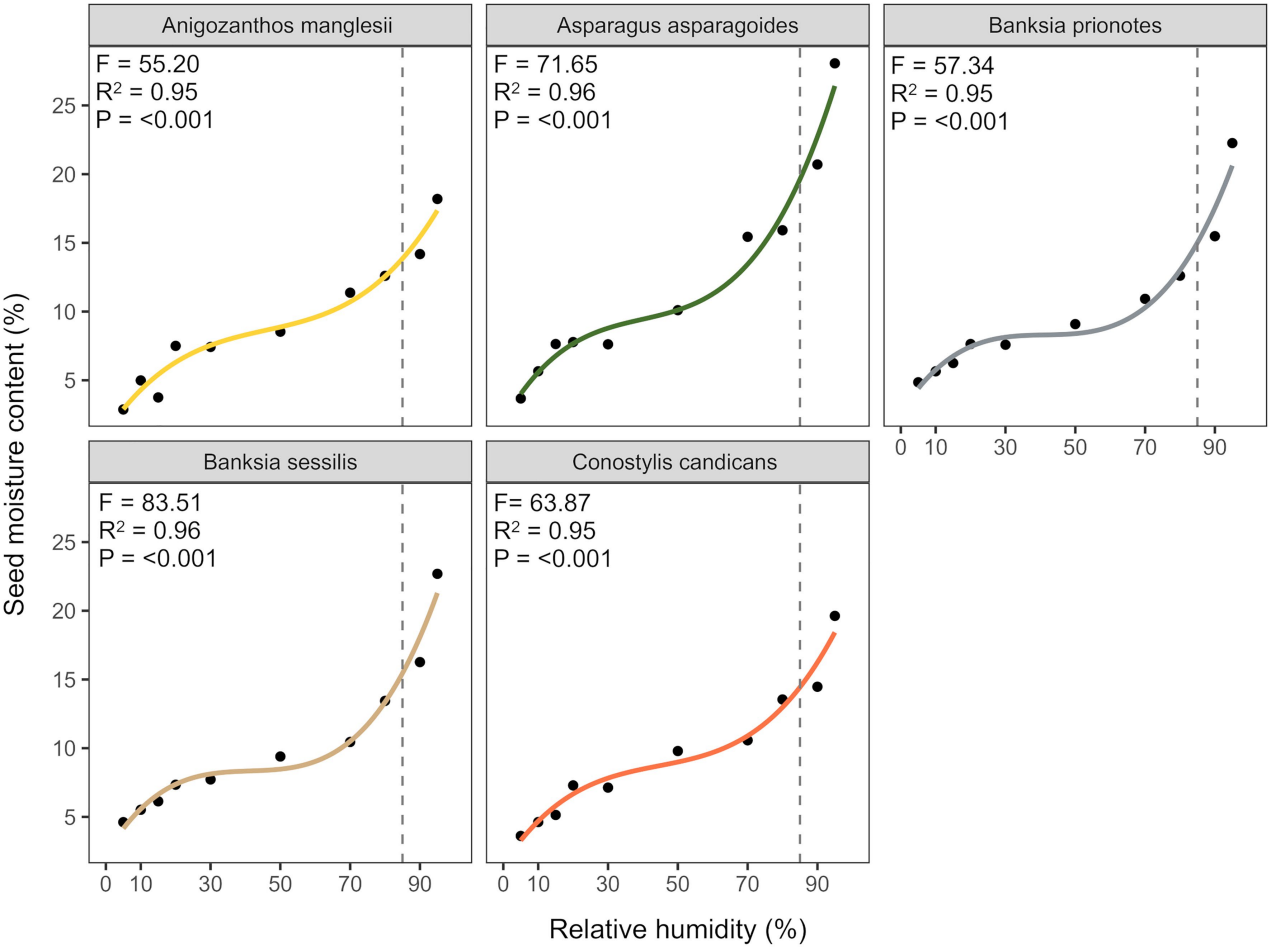
Fitted isotherms of the five study species using cubic polynomial models describing the relationship between seed moisture content and relative humidity of seeds, at 20°C.

There was a sigmoidal relationship between seed moisture content and relative humidity, with patterns in seed hydration effectively described by cubic polynomial models for all species (Figure 1: *R*^2^ = 0.95–0.96). Seeds of *A. asparagoides* had the highest moisture content at 85% RH, at ~20% moisture content, with all other species reaching c. 15% moisture content at 85% RH.

### Seed Moisture Content of Soil Stored Seeds

**Figure 2:**
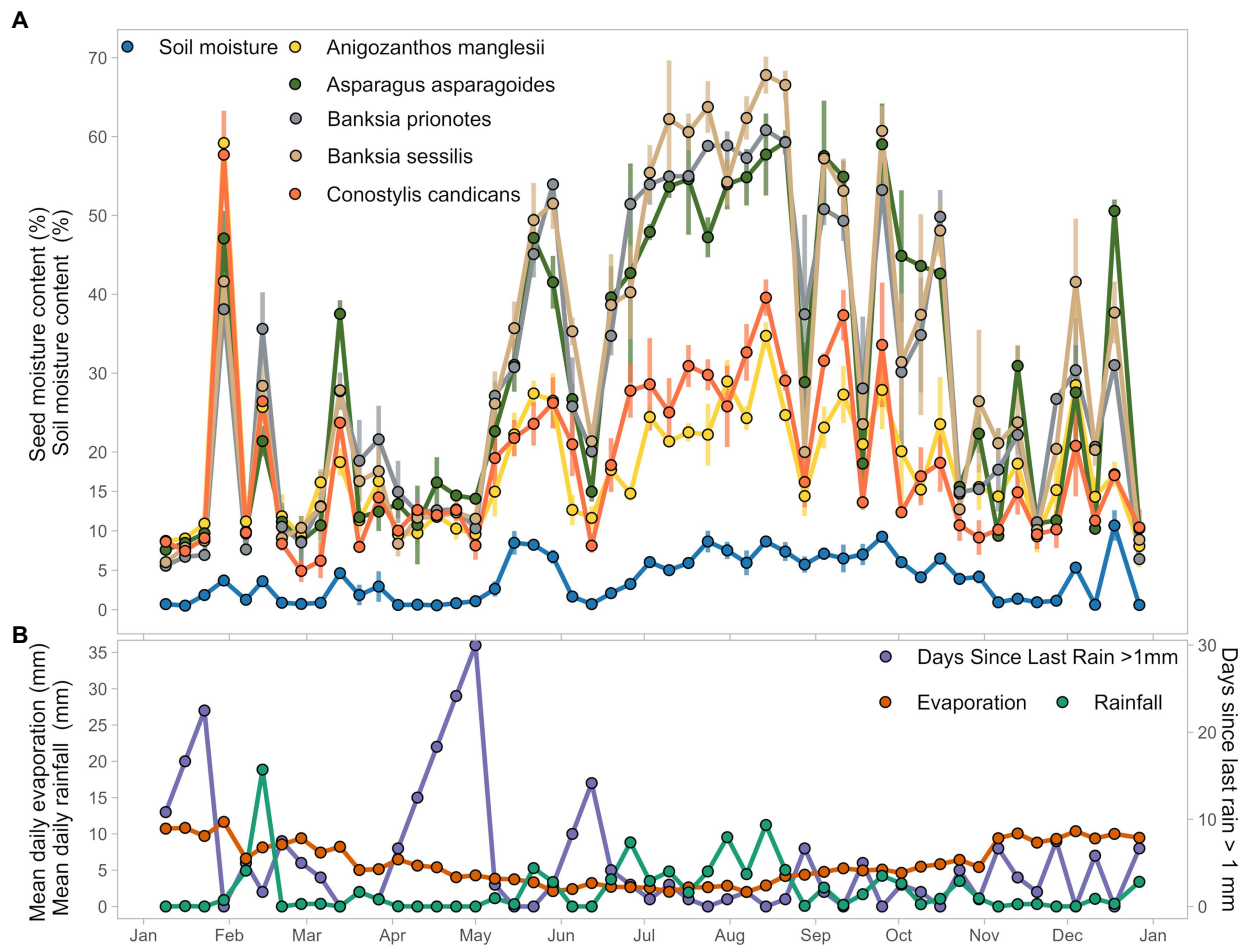
Seed and weather data visualized. Seeds were collected weekly for 51 weeks for the year of 2017. **(A)** Plotted mean seed moisture content, averaged from three replicates for each collection date, and mean soil moisture content averaged from three replicate samples each collection date. **(B)** Mean daily pan evaporation and mean daily rainfall for the 7 days preceeding the seed collection date, and number of days since last rain (>1 mm of rain) over the course of 2017. All weather data extracted from Bureau of Meteorology taken from Perth metro weather station and derived products (Bureau of Meteorology, 2020).

Across the 51 weeks of burial in soil, relative seed moisture content differed between species, but the patterns of hydration and dehydration were broadly consistent across all species. For example, seed moisture content increased rapidly after a rainfall event during the last week of January across all species, from the 8–10% measured in the first 3 weeks, to 40–60% (Figure 2A). Seed moisture content then decreased to 9–11% in the first week of February but increased again following rainfall in the next week to 20–40% across species. A similar rapid wetting and drying event was evident during and immediately following a rainfall event in March (Figures 2A,B). After June 12th, seed moisture content remained relatively high (17–50% across all species) until October 30th (Figure 2A). This period coincided with consistent rainfall, lower evaporation, and uniformly high soil moisture content (Figure 2B).

**Figure 3:**
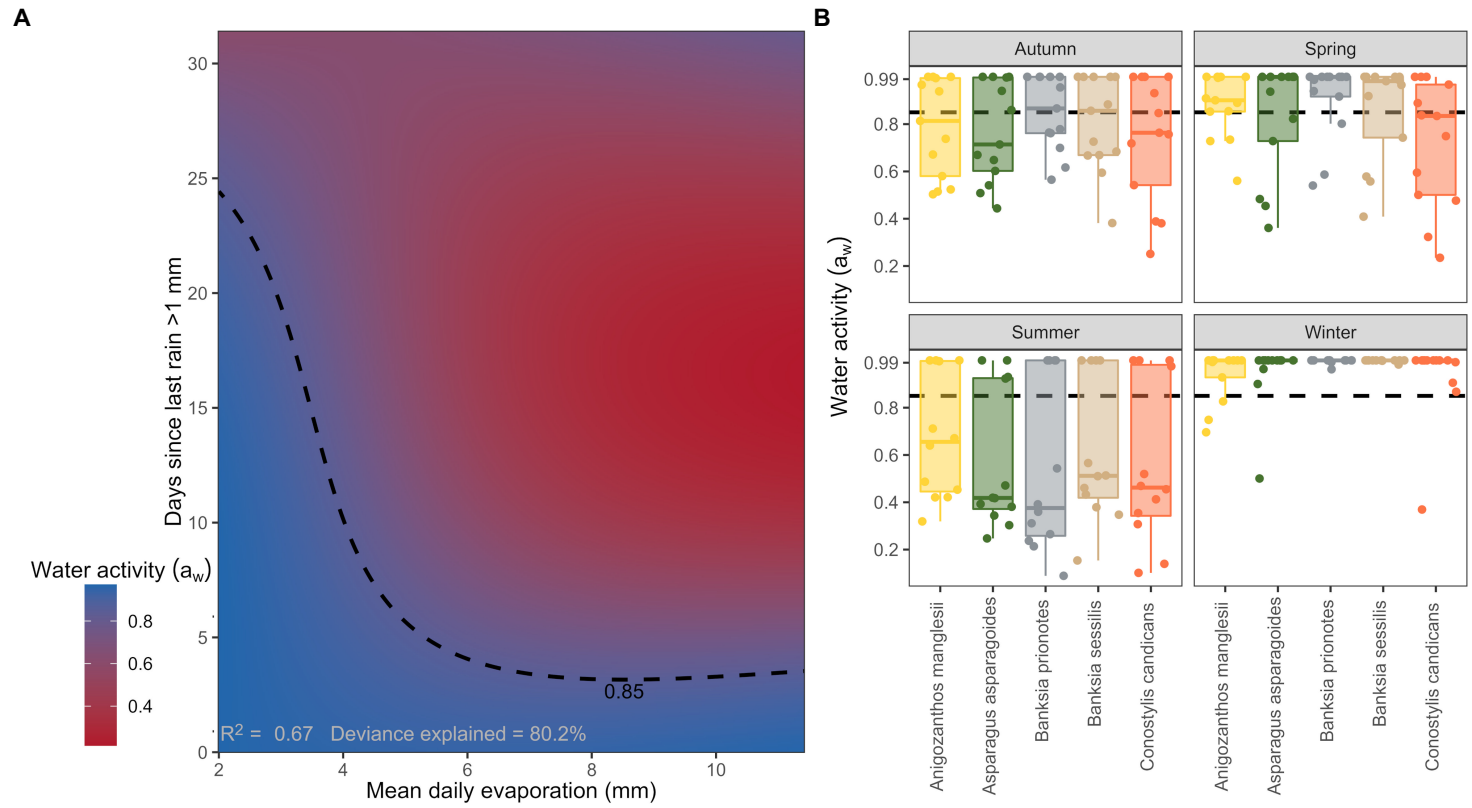
**(A)** Modelled interactive relationships between seed water activity for seeds within in the soil seed bank, mean daily evaporation, and days since last rain >1 mm. Dashed contour line indicates the threshold water activity of 0.85 *a*_w_. **(B)** Patterns of water activity for each individual species for the year of 2017 stratified by season. Dashed line indicates the hydration threshold where seeds are >0.85 *a*_w_.

The best performing model of the weather variables as drivers of seed water activity described ~67% of the variance in water activity, with an interactive function associated with the relationship between mean daily pan evaporation and the number of days since the last rain event >1 mm (Table 2; Figure 3A). Differences in individual species responses were important (Figure 3B) but were not included in the top model (Table 2).

**Table 2:** Top generalised additive models (GAMS; ΔAICc < 2) from full subsets analysis describing the patterns in seed water activity.

Model	AICc	edf	ΔAICc	ωAICc	*R* ^2^	Deviance explained
Mean daily evaporation * Days since last rain	−1389.34	7.84	0	0.52	0.67	80.2
Mean daily evaporation * Days since last rain + Species	−1389.17	11.85	0.16	0.48	0.69	81.3%

*Akaike’s Information Criterion corrected for small sample size (AICc), estimated degrees of freedom (edf), and difference from lowest reported AICc (ΔAICc), AICc model weights (ωAICc), variance explained (R^2^), and deviance explained are reported for model comparison*.

Seed water activity was highest when daily average evaporation was low and there were less than 25 days since the last rain event (Figure 3A). Seed water activity dropped below 0.85 *a*_w_ during extended periods (i.e., >25 days) without more than 1 mm of rain. If rainfall had occurred within the previous 5 days, seed water activity remained high (>0.85 *a*_w_) even if mean daily evaporation was high (i.e., 10 mm; Figure 3A).

Seed water activity was the lowest across all species during the summer months (December–February). Median seed water activity ranged from ~0.65 *a*_w_ for *A. manglesii*, to less than 0.40 *a*_w_ in *B. prionotes* during summer. Seed water activity varied considerably more during the autumn months (March through to the end of May), but for three out of the five species, median seed water activity remained below 0.85 *a*_w_ throughout. Seeds of *A. asparagoides* had the lowest median water activity during autumn ~0.70 *a*_w_. During winter (June through to the end of August) seed water activity was uniformly high, with ~0.99 *a*_w_ for all species. The months of spring (September through to the end of November) were similar to autumn whereby seed water activity varied considerably. However, it was evident that median seed water activity was generally higher (by 0.1–0.2 *a*_w_) in spring than in autumn, ranging from ~0.85 *a*_w_ for *C. candicans* up to 0.99 *a*_w_ for *B. prionotes* (Figure 3B).

## Discussion

In this study, we aimed to model potential drivers of seed hydration dynamics in the soil across a 1 year period to identify and predict risks of seed mortality during aseasonal fire. Our previous studies demonstrated that while seeds are dry, their likelihood of surviving temperatures associated with fire is much greater, with lethal temperatures of seeds exceeding 120°C at water contents equivalent to 0.5 *a*_w_ (Tangney et al., 2019; Table 1). The lethal temperatures of hydrated seeds (*a*_w_ ≥ 0.85) are much lower (c. 31–38°C lower for our study species, Table 1). The results of this present study show that seed moisture contents were sufficiently high (i.e., ≥ 0.85 *a*_w_) so as to have created an increased risk of mortality if a fire had occurred during an almost continuous period between May and November of the study period (i.e., 2017). The period of apparent increased risk of seed mortality from fire during vulnerable periods in winter and spring is in line with theory and evidence that suggests that susceptible plants may have a reduced recruitment following spring fires within these Mediterranean-climate type ecosystems (Miller et al., 2019, 2020a,b; Tangney et al., 2020). Further, the likelihood that fires that occur during periods of elevated seed moisture content may increase the risk of seed mortality potentially favours those species that produce seeds that remain dry throughout the year, such as seeds with water impermeable seed coats. Seeds with impermeable coats are defined as possessing physical dormancy and are commonly found in the Fabaceae and the Rhamnaceae (Turner et al., 2005), among others. Fires during periods when water permeable seeds are hydrated could contribute to a change in species composition towards these physically dormant species.

Within our study system, our models of the interaction between days since last rain and evaporation were able to describe 67% of the week-to-week variation in seed water activity. The models indicate that when mean daily pan evaporation is low and there has been more than 1 mm of rain in the past 25 days, there is a high likelihood that soil-stored seeds (with permeable seed coats) will be wet (i.e., ≥0.85 a_w_). If, however, average daily evaporation is ~4 mm, the window of time over which seeds remain wet reduces to just 8 days following a rain event of >1 mm, and further reduces to just the 5 days following rain >1 mm if the average evaporation is ≥5 mm. This simple metric, based upon the prevailing weather conditions, offers the potential to define those periods during which seed hydration conditions within the soil seed bank are such that the risks of seed mortality during fire are lessened. Periods when seeds are dry likely correlates with periods of elevated fire risk, as soils are drier and fuels that contribute to fire spread are likely to be more available.

Seeds imbibe moisture from their surrounding medium in the form of both liquid and vapour phase water (Wuest, 2007). We found that seed water activity was, on average, highest in winter and spring, and lowest in summer and autumn. During the winter and spring months, regular rainfall and low evaporation rates provided suitable, and relatively constant, levels of available soil moisture for seeds to remain hydrated, but as the time since rain increased and evaporation rates increased through the summer months, the soil moisture conditions became such that seeds were mostly in a dry state. Following long dry periods, rapid imbibition of seeds can evidently occur even upon relatively small rain events, as occurred in January 2017, where seed moisture quickly rose, despite only 6 mm of rain recorded in the week to 31st January. This rapid hydration is due to low water potentials in the dry seeds and the matric and osmotic pressures quickly drawing available soil water into seed tissues (Hadas, 1977). However, seeds dried equally rapidly during summer months, and we did not determine seed moisture contents during summer that exceed our threshold of increased risk of seed mortality (i.e., 0.85 a_w_).

The minimal differences in seed hydration between the different species throughout 2017 suggests that these results likely represent the generalised hydration pattern of many Banksia woodland seeds that have water permeable seed coats and spend some portion of their lifecycle in soil seedbanks. Whilst the wetting and drying patterns may be similar amongst species, interactions between seed hydration status and lethal temperatures influence species disproportionately. For example, dry seeds of *A. asparagoides*, a species native to South Africa but invasive across temperate Australia including within Banksia woodlands (Morin and Scott, 2012), have a lethal temperature lower than the examined native species while dry. The *A. asparagoides* seeds thus have a disproportionately greater risk of mortality during fire, and fires while seeds are dry may impede post-fire recruitment (Keeley, 2006), a factor that may be useful for reducing the spread of this invasive species. However, the differences in lethal temperatures between species greatly reduces when they are wet, meaning the relative advantage in survivability maintained by seeds of the natives during historical (dry) fire seasons is lost during burns under high soil moisture conditions.

Two of the species examined (*B. prionotes* and *B. sessilis*) commonly maintain canopy seed banks *via* the storage of seeds within woody fruits (serotiny; Lamont, 1991). Serotiny provides seeds stored within the woody fruits increased protection from fire temperatures, as well as from ambient moisture (Lamont et al., 1991; Huss et al., 2019), with seeds typically released from the fruits after fire (Lamont et al., 1991). However, within our study system of the Banksia woodlands of the Perth region, both *Banksia* species are weakly serotinous and release seeds in the absence of fire – annually and shortly after maturation – into the soil seed bank (Cowling and Lamont, 1985). Thus, the seeds of these species, despite originating from plants with serotinous characteristics, are still exposed to fire temperatures in, or on the surface of soils, if the seasonal timing of fire follows that of seed dispersal. Flowering in both species begins in early autumn and extends through winter, with seeds being dispersed during late spring and summer. Seeds will remain in the soil seed bank until conditions are suitable for germination or until the loss of seed viability, which may occur within 4–8 months following release (Miller et al., 2021). Their seeds are able to tolerate high temperatures while dry (e.g., 131.6 and 144.0°C, respectively; Table 1), which may limit their mortality in soil seed banks during naturally occurring fires. But, like the other species in our study, seeds of these *Banksia* spp. will be exposed to greater risk of mortality from aseasonal fire during periods of high soil moisture.

In this study, seeds were buried under 2 cm of loose soil and a shallow layer of leaf litter. As such, soil moisture may be lost more quickly compared to the rate of moisture loss deeper within the soils, or under heavier litter (Tromp-van Meerveld and McDonnell, 2006). While the majority of seeds are buried within the top 2 cm of the soil seed bank, deeper burial, or more leaf litter, may potentially extend the number of days required for seeds to dry below our threshold of 0.85 *a*_w_. Moisture loss from soils is reduced further by the presence of ground cover, including shrub and canopy cover, leading to a reduction in evaporation of moisture from soils (Lamb and Chapman, 1943). Consequently, seeds within soils under shrub or canopy cover may potentially remain hydrated for periods significantly longer than seeds measured in this study. But soil burial depth of seeds may also influence seed mortality *via* increased insulation from fire temperatures as soil depth increases. Soils are strong insulators against heat, able to greatly reduce the temperatures experienced deeper within the soil profile (Tangney et al., 2020a). The insulative properties of soil increases further while soils are wet as available moisture quenches the thermal energy directed into the soil from the fire, converting liquid water into steam and, thus, dampening temperatures (Stoof et al., 2011). Nevertheless, in cases where fuels are dry enough to actively combust while soils are still wet, soil temperatures can rapidly increase once soil moisture is boiled off (Aston and Gill, 1976; Tangney et al., 2018). Therefore, if seeds are wet while surface fuels are dry, seeds stored within the soil seed bank may still be at increased risk of mortality.

We found that days since last rain and mean daily evaporation best predicted seed hydration status, more so than soil moisture and mean daily rainfall. This result was unexpected as soil moisture sampled from adjacent to the seed burial area was anticipated to be closely correlated with seed moisture (Wuest, 2007). Whilst our study design may have influenced the strength of potential contributions of soil water deeper than 2 cm due to the plastic trays potentially influencing water vertical movement of water towards the soil surface, >50% of seeds within the seedbank reside at depths less than 2 cm and the conditions within the trays did resemble that of the upper soil profile (Rokich et al., 2016). Nevertheless, the model described was a powerful predictor of seed moisture, and it has the additional benefit of using commonly reported metrics that are easily calculated from nearby weather stations, rather than requiring more complex *in situ* soil sampling. This study was carried out in an ecosystem with characteristically sandy soils which contains little organic material, limiting the water holding capacity of (Ritchie et al., 2021). The relationships between seed hydration status and weather variables here may likely vary in systems with different soil texture, organic matter content, and moisture retention characteristics, thus influencing how the model predictions perform.

## Conclusion

During fire, elevated soil temperatures can lead to mortality of seeds stored in contact with soils. Fires that occur while seeds buried within soil seed banks have high moisture contents may further enhance seed mortality. In this study, we have described a model that informs when seeds are most likely to be at greater risk to mortality during fire, *via* simply calculated weather metrics. Our model was able to capture ~67% of the variation in seed water activity across the year, providing key insights into when seeds are most likely to be hydrated, and thus exposed to increased risk of mortality from elevated soil temperatures during fire. Seeds were most likely hydrated shortly after rain, when pan evaporation was low. Seeds remain hydrated following rainfall that occurred within the previous 5 days, even when mean daily evaporation was high (i.e., 10 mm). Seed hydration status was sufficiently high (i.e., ≥0.85 a_w_) so as to have created an increased risk of mortality if a fire had occurred during an almost continuous period between May and November of the study period (of 2017). There were brief windows when seeds may have been in a dry state during early winter and late spring, and also when they may have been in a wet state during summer and late autumn. Fires that occur while seeds are hydrated may increase seed mortality in soil seed banks, and repeated unseasonal fires may lead to significant community shifts where species that produce water permeable seeds are lost from the ecosystem.

## Data Availability Statement

The raw data supporting the conclusions of this article will be made available by the authors, without undue reservation.

## Author Contributions

RT, BM, and DM all equally contributed to the ideas and design of this manuscript and editing of the manuscript. RT led collection, analysis of the data, and the writing. All authors contributed to the article and approved the submitted version.

## Funding

Support for this work was received from Australian Research Council (ARC) Linkage Grant ARC Linkage Grant LP180100741 to BM, supporting RT.

## Conflict of Interest

The authors declare that the research was conducted in the absence of any commercial or financial relationships that could be construed as a potential conflict of interest.

## Publisher’s Note

All claims expressed in this article are solely those of the authors and do not necessarily represent those of their affiliated organizations, or those of the publisher, the editors and the reviewers. Any product that may be evaluated in this article, or claim that may be made by its manufacturer, is not guaranteed or endorsed by the publisher.
